# Green adherent degradation kinetics study of Nirmatrelvir, an oral anti-COVID-19: characterization of degradation products using LC–MS with insilico toxicity profile

**DOI:** 10.1186/s13065-023-00928-z

**Published:** 2023-03-17

**Authors:** Sara I. Aboras, Hadir M. Maher

**Affiliations:** Pharmaceutical Analytical Chemistry Department, Faculty of Pharmacy, University of Alexandria, Elmessalah, Alexandria 21521 Egypt

**Keywords:** Nirmatrelvir, Degradation, Kinetics, LC–MS, Greenness

## Abstract

The SARS-CoV-2 virus sets up a global catastrophe, and countries all around the world made significant efforts to halt the spread. Nirmatrelvir (NMV) was lately approved by the FDA as a safe and well-tolerated oral direct-acting antiviral medication for SARS-CoV-2 virus infection. Therefore, a fast completely validated stability indicating method was established-for the first time- for NMV determination. The study used NaOH, HCl, neutral, H_2_O_2_, and sunlight to test NMV stability under various stress conditions followed by kinetics degradation investigation and derivation of Arrhenius plot. The analysis was performed using Agilent Zorbax Eclipse-C18 column (5 µm, 4.6 × 250 mm) with a mobile phase consisting of acetonitrile: 50 mM ammonium acetate, pH = 5 (50:50, v/v, respectively) at a flow rate of 1.0 mL/min with 5 min run time. Diode array detector (DAD) was set at 225 nm to quantify NMV at the concentration range of 5–500 µg/mL with LOD and LOQ of 0.6 and 2 µg/mL, respectively. Method’s greenness was assessed using different metrics including Analytical Eco-Scale, Greenness Assessment Procedure Index, GAPI, and Analytical Greenness, AGREE. A thorough study of stress stability revealed that NMV was more susceptible to alkaline hydrolysis compared with acid hydrolysis. In contrast, it was found that NMV remained stable when subjected to oxidative, neutral, and sun-induced degradation conditions. Moreover, acid and alkali-induced hydrolysis were found to follow pseudo first order kinetics. Consequently, the half lifetime of the studied degradation conditions at room temperature were calculated using the Arrhenius plot. The mechanism of the degradation pathways under stress circumstances was proposed using LC–MS-UV. Toxicities of the proposed degradation products were assessed using ProTox-II, along with the parent medication NMV, and were shown to be hardly hazardous.

**Supplementary Information:**

The online version contains supplementary material available at 10.1186/s13065-023-00928-z.

## Introduction

Coronavirus disease 2019 (Covid-19) with its severe acute respiratory syndrome coronavirus 2 (SARS-CoV-2) has continued to be a concern to world health. Elderly people, smokers, or those with underlying clinical diseases including cardiovascular diseases, diabetes, obesity, or cancer, are at a higher risk of severe Covid-19 and its consequences compared with normal people. It was reported that people with co-existing diseases are almost two times more probable to develop severe Covid-19 infections with a mortality rate five times more than those without such underlying diseases [1–6].

There is a need for safe and effective oral Covid-19 therapies that can accelerate the recovery and prevent the progression of the infection to more serious symptoms including hospitalisation, and probably death. Such therapies would aid the health care system to manipulate the problems of overcrowded hospitals with inadequate facilities. Monoclonal antibodies were widely used for non-hospitalized individuals with mild-to-moderate Covid-19 infections. However, they have several drawbacks, such as the requirement for clinical investigation and the possibility of diminished efficacy against developing SARS-CoV-2 variants [7].

Nirmatrelvir (NMV) is an antiviral drug that targets the SARS-CoV-2 enzyme, 3-chymotrypsin–like cysteine protease. NMV effectively inhibits the enzymatic activity with subsequent inhibition of virus multiplication across a wide range of coronaviruses. Being a CYP3A4 substrate, it was found that coadministration of 300 mg NMV with a low dose (100 mg) of ritonavir, a CYP3A4 inhibitor, twice daily enhances NMV pharmacokinetics [8, 9]. In December 2021, Pfizer’s Paxlovid^®^ (nirmatrelvir tablets and ritonavir tablets, co-packaged for oral use) has received an emergency use authorization (EUA) from the US Food and Drug Administration for the treatment of mild-to-moderate COVID-19 in adults and paediatric patients with positive results of direct SARS-CoV-2 testing and who are at a high risk of progressing to severe COVID-19, including hospitalisation or death [10].

Drug stability affects product safety and efficacy since degradation products (DPs) may cause a loss of potency and result in potential toxic effect. Accordingly, chemical and physical stability are important to ensure the quality and safety of active ingredients [11].

To the best of our knowledge, only one fully validated method was published for analysing NMV in human plasma along with ritonavir using LC–MS/MS [12]. Another article has been recently published for the characterization of NMV’s DPs using LC-UV-HRMS^n^ [13]. Although this published work [13] provided detailed postulation of degradation pathways, it did not cover any quantitative aspects concerning method validation, determination of the intact drug in presence of its DPs, or studying the degradation kinetics. Considering the high expenses of LC–MS/MS instrumentation besides its need to high expertise in its operation, and its unavailability in many analytical labs, HPLC–DAD is considered a simple alternative for quantitative applications. *To the best of our knowledge*, neither a validated HPLC–DAD method for the determination of NMV nor information about the degradation kinetics was found in the literature so far. *This work aims at developing and validating a simple *stability-indicating HPLC–DAD method-*for the first time*-for the determination of NMV in presence of its DPs generated under different stress conditions. The application of this method was extended to studying *the degradation kinetics* of NMV which is extremely important in determining the order of the reaction along with the half lifetime using Arrhenius plot. Moreover, it is a crucial target to study the safety profile of drugs and their DPs, accordingly insilico toxicity prediction software was used in this work with NMV and its DPs which adds to *the novelty of the proposed study*. In addition, the eco-friendlessness of the validated HPLC–DAD method was assessed using different greenness tools.

## Experimental

### Materials and reagents

Nirmatrelvir (NMV) was kindly supplied from KU Leuven Research & Development, Belgium with approximate purity of 94%. El-Nasr Chemical Industry Company in Egypt provided Analytical quality grade hydrochloric acid (HCl) and sodium hydroxide (NaOH). Hydrogen peroxide (H_2_O_2_), 50%, was also obtained from El-Nasr Chemical Industry Company.

Both HPLC- grade acetonitrile (ACN) and methanol (MeOH) were purchased from Baker's, Ireland. Water was deionized and filtered using Ultrapure PTFE syringe filters, 0.2 µm pore size.

### Apparatus

The HPLC–DAD system consisted of Agilent 1200 series (auto-injector, quaternary pump, vacuum degasser and diode array with multiple wavelength detector G1315 C/D and G1365 C/D connected to a computer loaded with Agilent ChemStation Software (Agilent Technologies, Santa Clara, CA, USA).

Shimadzu^®^ UFLC series (Shimadzu Corporation, Kyoto, Japan) was used with its LC–MS-UV system hyphenated with two detectors: UV detector SPD-20A and MS detector LCMS-2020 with mass range of m/z 10 to 2000. The detector was supplied with single quadrupole system and two ion sources ESI and APCI (Shimadzu Corporation, Kyoto, Japan). The scan rate was up to 15,000 amu/sec. A Shim-pack XR-ODS II^®^ C18 analytical column (100 × 3 mm, 2 µm) was used as the stationary phase (Shimadzu, Japan).

### Chromatographic conditions

#### HPLC–DAD

The separation was performed on an Agilent Zorbax Eclipse-C18 analytical column (250 × 4.6 mm, 5 µm). The mobile phase system consisted of solvent A (50 mM ammonium acetate, pH = 5) and solvent B (ACN) in an isocratic elution made of 50:50, v/v, respectively. Detection wavelength was set to 225 nm. A flow rate of 1 mL/min and 20-µL injection volume were used. The entire run time was 5 min.

#### UFLC-MS-UV

A Shim-pack XR-ODS II^®^ C18 analytical column (100 × 3 mm, 2 m), Shimadzu, Japan, was employed for the separation. The mobile phase used was the same as that for HPLC–DAD. Detection was performed using an electrospray ionisation (ESI) interface set to the selected ion monitoring (SIM) mode with positive and negative polarity. The flow rate was kept at 0.4 mL/min. Injection volumes of 10 µL were used all over the study. The run time was 7 min.

### Preparation of stock standard solutions and construction of calibration graphs

Stock solutions of NMV 1000 µg/mL were prepared in HPLC-grade methanol. The solutions were kept refrigerated at 0 °C. Dilution of the stock solutions using deionized water was performed to get working standards within the concentration range 5–500 µg/mL. Each concentration was injected in triplicate into the HPLC–DAD system under the optimized conditions using 20 µL injection volume. The peak area of NMV recorded at its R_t_ of 4.01 ± 0.01 min was related to each concentration to get the regression equation and the calibration graph.

Analytical method validation of NMV was appraised for accuracy, precision, linearity, specificity, limit of quantitation and robustness in agreement with ICH guidelines [14].

### Forced degradation and stability-indicating study

Forced degradation studies were done using separate volumes of 1 mL of NMV stock solution (1000 µg/mL). Following degradation, the solutions were then completed to volume in a series of 10-mL volumetric flask with deionized water to reach a final concentration of 100 µg/mL.

### Acidic and alkaline hydrolysis

NMV solutions were treated with 1 mL volumes of 1 M HCl or 0.1 M NaOH, for acidic and alkaline hydrolysis, respectively. Solutions were then heated in a thermostated water bath kept at 90 ℃ for 1 h for acidic hydrolysis or at 60 °C for 30 min for alkaline hydrolysis. After the specified time intervals, the solutions were cooled then neutralized to pH 7. Degraded solutions were then diluted with deionized water in 10-mL volumetric flasks to reach a specified final concentration of 100 µg/mL.

### Neutral hydrolysis

Volume of 1 mL of deionized water was added to NMV solutions. The mixture was heated in a thermostated water bath at 80 ℃ for 1 h, then cooled and completed to volume as mentioned above.

### Oxidative degradation

NMV solution was treated with 1 mL of hydrogen peroxide (30% v/v), heated in a thermostated water bath at 60 ℃ for ½ h. The solutions were then cooled and diluted as above.

### Photolytic degradation

Photo-stability study was performed by exposing NMV stock solution (1 mL of 1000 µg/mL) to sunlight during daytime for 4 h. After the specified time, the volume was completed as shown above.

### Kinetics investigation and Arrhenius plot

Degradation of NMV in an alkaline medium using 0.1 M NaOH and in an acidic medium with 2 M HCl was studied as a function of temperature.

For alkaline hydrolysis, volumes of 1 mL 0.1 M NaOH were separately added to NMV stock solution (1 mL of 1000 µg/mL) and mixed well. The solutions were kept in a thermostated water bath at different temperatures (50, 60 and 70 °C). After specified time intervals (0.5, 1, 1.5 and 2 h), the solutions were taken, left to cool, then neutralized to pH 7 before being diluted with the mobile phase to reach a final concentration of 100 μg/mL of NMV.

For acidic hydrolysis, separate volumes of 1 mL 2 M HCl were added to 1 mL of NMV stock solution and mixed well. The solutions were kept in a thermostated water bath at different temperatures (60, 70 and 80 °C). After the specified time intervals, 0.5, 1, 1.5 and 2 h, the solutions were then neutralized and diluted with the mobile phase to reach final concentrations of 100 μg/mL of NMV.

The regression equation for NMV determination was then used to determine the recovered concentration of the intact drug in the treated solutions. By monitoring the decline in NMV concentration over time, the degradation kinetics was determined.

### LC–MS-UV study of NMV and its degradation products

LC–MS-UV was used to elucidate the structure of NMV DPs’ under alkaline and acidic stress conditions. Alkaline-induced DPs were generated using 0.1 M NaOH, 25 °C, 4 h or 0.1 M NaOH, 100 °C, 1 h, for partial and complete hydrolysis, respectively. Acidic-induced DPs were generated through partial and complete degradation of NMV using 1 M HCl at 90 °C for 1 h and 2 M HCl at 100 °C for 4 h, respectively. Degraded solutions were neutralised before being diluted to 100 μg/mL of NMV. NMV degraded solutions were initially injected into the LC–MS-UV system for the purpose of identification using the settings listed under section “Chromatographic conditions”. Using ESI with a mass range of 100 to 1000 m/z, the peaks of NMV and its probable DPs were monitored in both positive and negative scan mode.

### In-silico toxicity studies

Using the Pro Tox-II web server tool, the toxicity of NMV and its main DPs was predicted in silico. Leave-one-out cross-validation was used to validate the ProTox-II acute toxicity model. The parameters of ProTox-prediction II had been adjusted to enhance toxicity class and LD50 prediction hit rates. Generally, after being exposed to a chemical, the median lethal dose (LD50) was calculated [15].

### Greenness of the method

Different methodologies were used to assess the degree of greenness of the HPLC–DAD involved in the study. These methodologies include the Analytical Eco-Scale tool, the green analytical procedure index (GAPI), and the Analytical greenness metric (AGREE).

## Results and discussion

A green stability-indicating assay method that can separate NMV and its DPs from each other is required to conduct a detailed kinetic degradation investigation of NMV. DPs should be structurally characterized to define their in-silico toxicity. As a result, Pro Tox- II was used to determine their potential toxicity. Finally, the proposed HPLC–DAD method’s degree of greenness was evaluated using different tools.

### Optimization of the HPLC–DAD chromatographic conditions

Two reversed phase columns were tried, namely Agilent Zorbax eclipse-C18 (150 × 4.6 mm, 5-µm) and Agilent Zorbax eclipse-C18 (250 × 4.6 mm, 5-µm). The separation efficiency of the two columns was evaluated using mobile phases of ACN and ammonium acetate, pH = 5 in the ratio of 50:50, v/v, respectively. Experimental trials revealed that the longer column achieved better separation between NMV and its DPs. Thus, the longer column, of 250 mm length, was used for the study.

**Fig. 1:**
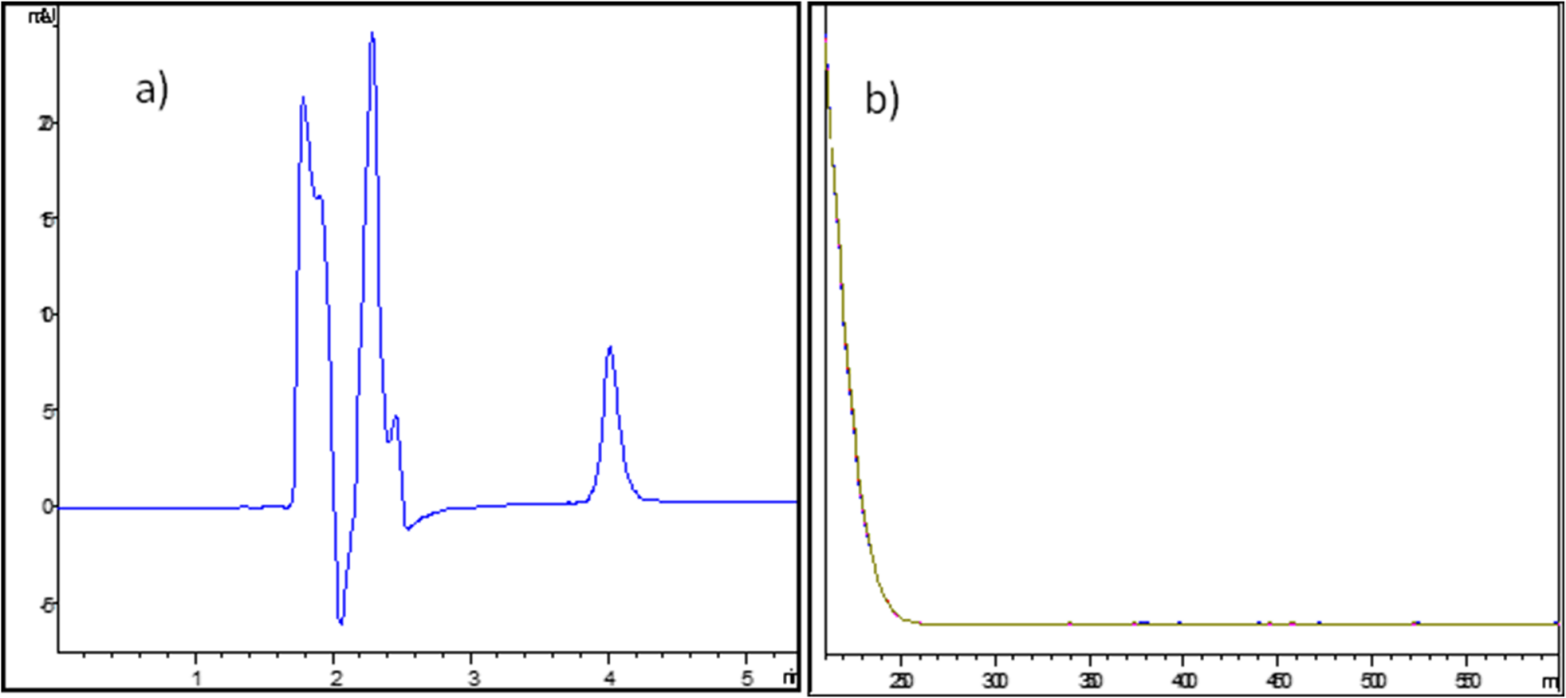
HPLC DAD chromatogram of 10 µg/mL NMV (**a**), and the corresponding UV spectrum (**b**), using the proposed HPLC–DAD method

Different pH values of the aqueous phase were tried (3, 5 and 7). It was found that pH 3 and 5 gave better peak shape since the pka value of NMV is 7.1 [16]. While pH 3 and 5 gave similar results concerning peak area, resolution and tailing factor, pH 5 was chosen as it is more suitable with respect to the buffer capacity of ammonium acetate [17]. Both MeOH and ACN were tried as organic modifiers. ACN produced more symmetrical and sharper peaks compared with MeOH, thus ACN was used in subsequent trials. Isocratic elution was suitable for the development of a stability indicating method of NMV with adequate resolution between NMV and its nearest DP. Pure peaks were confirmed by calculating purity factors using DAD and they were within the appropriate automatically calculated noise acceptable limit, Additional file 1: Fig. S1. The run time was 5 min, with a 4.01-min retention time for NMV determination and the detection wavelength was 225 nm which corresponded to NMV maximum wavelength as shown in Fig. 1.

### Forced degradation and stability-indicating study

NMV was subjected to different stress conditions, including acid, base, neutral, oxidative, and photo-degradation, as per ICH guidelines [14]. The proposed HPLC–DAD method was used to track the degradation of NMV. It is noteworthy to mention that the choice of degradation conditions is a matter of “trial and error” to get reasonable % degradation of the intact drug, 30–70%. The stability-indicating potential of the developed methods is then assessed by its ability to separate the intact drug from its DPs. Accordingly, studying the degradation kinetics requires the use of stress conditions which result in stepwise degradation of the intact drug. On the other hand, structure elucidation of DPs requires the use of violent degradation conditions resulting optimally in 100% degradation of the intact drug.

**Fig. 2:**
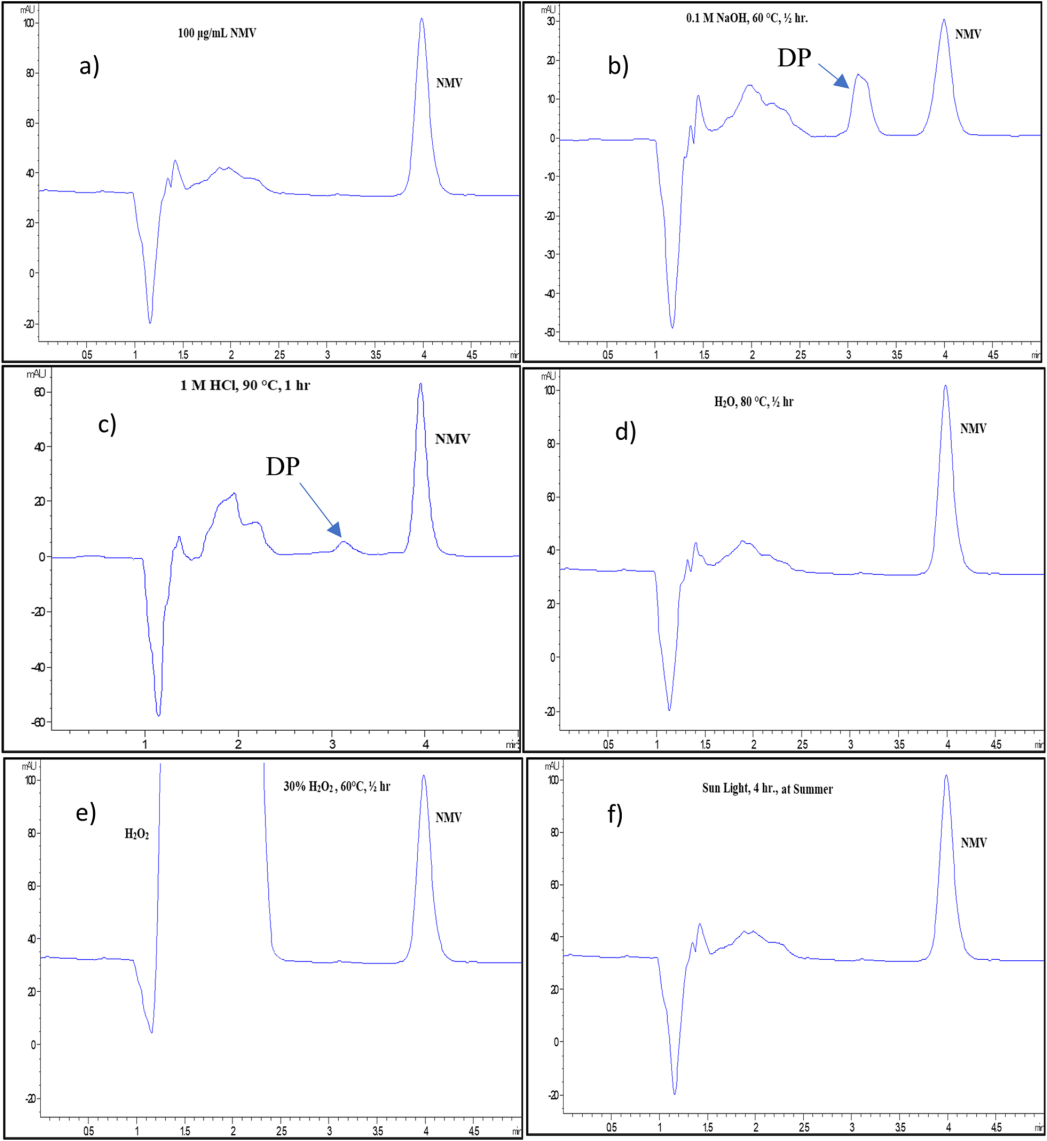
HPLC chromatograms of 100 µg/mL of NMV standard solution, (**a**), compared with different stress conditions: alkaline degradation (0.1 M NaOH, 60 °C, ½ h) (**b**), acidic degradation (1 M HCl, 90 °C, 1 h) (**c**), neutral degradation (H_2_O, 80 °C, ½ h) (**d**), oxidative degradation (30% H_2_O_2_, 60 °C, ½ h) (**e**), photo-degradation (day light, 4 h) (**f**)

Table 1 shows the different testing stress conditions that were tried during method development. Preliminary experiments showed that NMV remained stable with nearly 100% recovery under the following conditions: oxidative hydrolysis with 10% H_2_O_2_ at room temperature for 24 h, neutral hydrolysis with H_2_O, 60 °C, ½ h, and acidic hydrolysis using 0.1 M HCl at room temperature for 24 h. However, under alkaline degradation conditions, 0.1 M NaOH at 60 °C for ½ h, excessive degradation of NMV was noticed, with 39.56% recovery for NMV, Fig. 2. Further experimentation showed that NMV remained stable following the application of stronger oxidative/neutral degradation conditions, Table 1. On the other hand, stronger acidic conditions, 1 M HCl, 90 °C for 1 h, resulted in NMV degradation with 66.26% recovery. These findings revealed higher susceptibility of NMV to alkaline compared with acidic hydrolysis and that the drug was stable to oxidative, neutral, and sun light degradation conditions, as shown in Table 1, Fig. 2.

**Table 1:** Summary of the degradation behaviour of NMV using the proposed HPLC–DAD method

		Degradation Conditions	% Recovery
Alkaline degradation	Mild	0.1 M NaOH, 60 °C, ½ h	39.56
Acidic degradation	Mild	0.1 M HCl, RT, 24 h	98.85
	Strong	1 M HCl, 90 °C, 1 h	66.26
Neutral degradation	Mild	H_2_O, 60 °C, ½ h	100.21
	Strong	H_2_O, 80 °C, 1 h	100.24
Oxidative degradation	Mild	10% H_2_O_2_, RT, 24 h	99.65
	Strong	25% H_2_O_2_, 60 °C, ½ h	101.01
Sun light degradation		Direct sun light for 4 h	98.96

In conclusion, the proposed stability-indicating HPLC–DAD method enabled the separation of NMV from its DP under different degradation conditions. Peak purity was assessed by DAD, Additional file 1: Fig. S1.

### Validation of the proposed HPLC–DAD method

As a crucial step in developing analytical methods, system suitability parameters were determined to assess the chromatographic system's performance. The obtained results, shown in Additional file 1: Table S1, included resolution, tailing factor, column efficiency, and theoretical plates, and they all fell within FDA acceptance standards [18].

Different validation parameters were evaluated as per FDA guidelines [18] including linearity and range, detection and quantitation limits, accuracy and precision, specificity, robustness, and stability of solutions.

### Linearity and concentrations ranges

The linearity of the proposed HPLC–DAD method was evaluated in the concentration range of 5 to 500 µg/mL NMV and the linear regression equation Y = 4.14 + 1.10X was derived. Good linearity was shown by the high correlation coefficient (r = 0.9998). Additionally, the slope's RSD percent (Sb%) was determined to be 0.01 (less than 2%) indicating a small deviation around the slope. In addition, the small significance F (6.3 × 10^–8^) values indicated little scattering of the experimental data sets around the regression line [19, 20].

### Detection and quantitation limits

Limits of detection (LOD) and quantitation (LOQ) were evaluated using a signal-to-noise ratio of 3:1 for LOD and 10:1 for LOQ. Low LOD and LOQ values (0.6 and 2 µg/mL, respectively) of the proposed method provided evidence of its good sensitivity.

### Accuracy and precision

Three selected concentrations of NMV were analysed three times on the same day (intra-day) and on three successive days (inter-day). To assess the method's accuracy and precision, relative error (Er) and relative standard deviations (RSD) were calculated, respectively, Additional file 1: Table S2. The low values of Er and RSD illustrated the method's high accuracy and precision [19].

### Specificity

According to FDA guidelines [18], the specificity was evaluated by the method's capacity to successfully quantify NMV while being separated from its forced degradants with a reasonable resolution (more than 2). Additionally, NMV peak purity, assessed by DAD, was an index of NMV purity during its analysis with the potential degradants (stress degradation study), Additional file 1: Fig. S1.

### Robustness

The working wavelength (± 1 nm), mobile phase ratio (± 2%) and buffer pH (± 0.2 pH units) of the working chromatographic conditions were slightly altered to test the method's robustness. It was found that no significant changes were recorded on the measured responses or retention times, RSD% less than 2%, Additional file 1: Table S3.

### Stability of solutions

No chromatographic changes were observed for NMV standards when kept at room temperature for 6 h. Additionally, it was found that NMV stock solution, prepared in HPLC-grade MeOH, was stable when kept refrigerated at 0 °C for a week. This was supported by the absence of any foreign peaks besides unchanged peak area and retention times of NMV.

### Investigation of degradation kinetics and Arrhenius plot

**Fig. 3:**
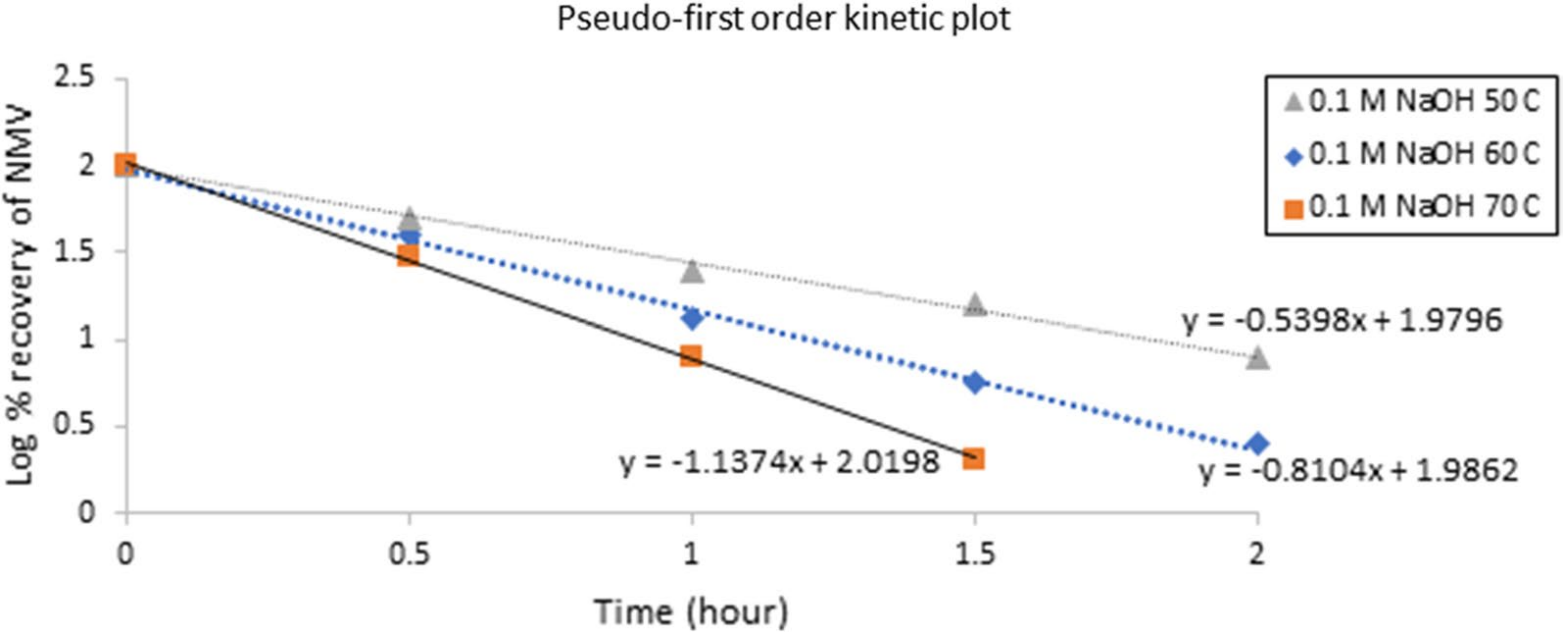
Pseudo first order Kinetic plot of NMV at different temperatures using 0.1 M NaOH

**Fig. 4:**
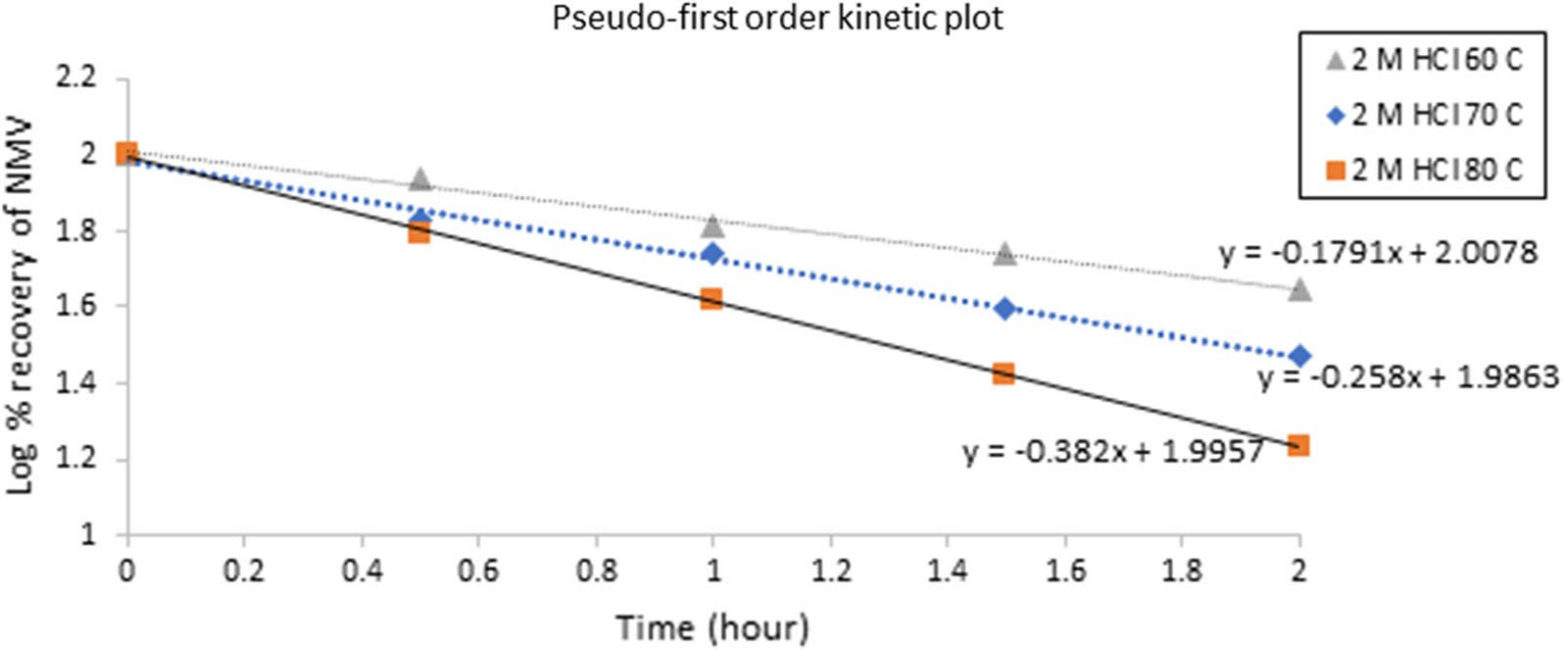
Pseudo first order Kinetic plot of NMV at different temperatures using 2 M HCl

The susceptibility of NMV to degradation in alkaline and acidic conditions paid our attention to extend the applicability of the proposed HPLC–DAD method to study the degradation kinetics, *a novel aspect in our study*. This was done by calculating the concentration of NMV remaining over a period (t) at different temperatures (50, 60, and 70 °C) and (60, 70 and 80 °C) for alkaline (0.1 M NaOH) and acidic (2 M HCl) degradation, respectively. The half-life (t_1/2_) and rate constant (K) of NMV degradation at the specified temperatures were calculated. In addition, the Arrhenius plot enabled the prediction of the on-shelf stability of NMV at ambient temperature. Figures 3 and 4 showed a linear decline in the log of the percent NMV remaining over time under the alkaline and acidic conditions, respectively. This indicated pseudo first-order degradation kinetics. The following equations were used [21].

$$\mathrm{log}\left(Ct\right)=\mathrm{log}\left(Co\right)- \frac{kt}{2.303}$$

$${t}_\frac{1}{2}=\frac{0.693}{{k}_{obs}}$$

where, C_t_ is NMV remaining concentration, after time (t), C_o_ is the initial concentration of NMV,

K, with negative sign, is the apparent first order rate constant.

For NMV alkaline hydrolysis, the (t_1/2_) values calculated at 70, 60 and 50 ℃ were found to be 0.28, 0.35 and 0.52 h, with corresponding observed reaction rate constants (k_obs_) of 2.46, 1.95 and 1.33 h^−1^, respectively. On the other hand, NMV acid hydrolysis resulted in (t_1/2_) values of 0.78, 1.11 and 1.72 h calculated at 80, 70 and 60 ℃, respectively with corresponding (k_obs_) values of 0.89, 0.62 and 0.40 h^−1^, respectively, Table 2.

**Table 2:** Pseudo first-order rate constants (K) and half-lives (t_1/2_) for the alkaline and acidic hydrolysis of NMV using different temperatures

Alkaline hydrolysis (0.1 M NaOH)		
Temperature/Strength	K (h ^−1^)	t_1/2_ (h)
70 ℃	2.46	0.28
60 ℃	1.95	0.35
50 ℃	1.33	0.52
Room temperature (25 ˚C) ^a^	1.22	0.56

Acidic hydrolysis (2 M HCl)		
Temperature/Strength	K (h ^−1^)	t_1/2_ (h)
80 ℃	0.89	0.78
70 ℃	0.62	1.11
60 ℃	0.40	1.72
Room temperature (25 ˚C)^a^	0.13	5.39

^a^Calculated by extrapolation of the Arrhenius plot

**Fig. 5:**
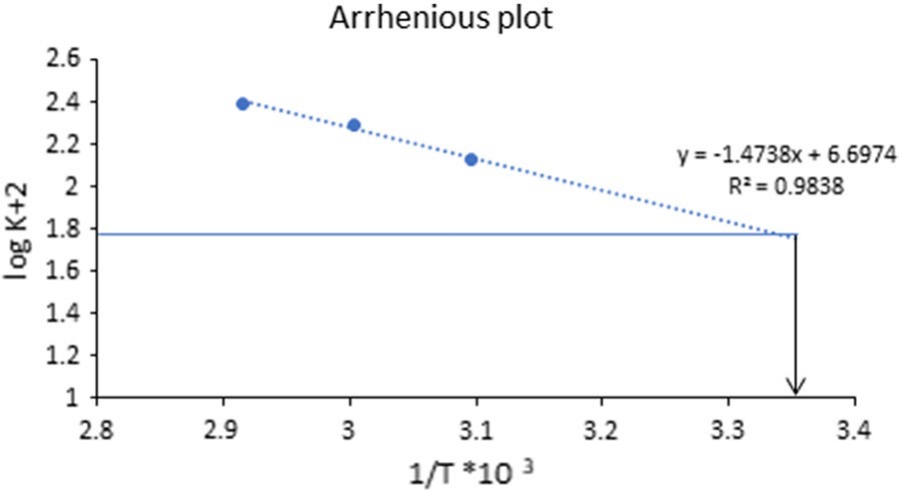
Arrhenius plot for NMV alkaline degradation using 0.1 M NaOH

**Fig. 6:**
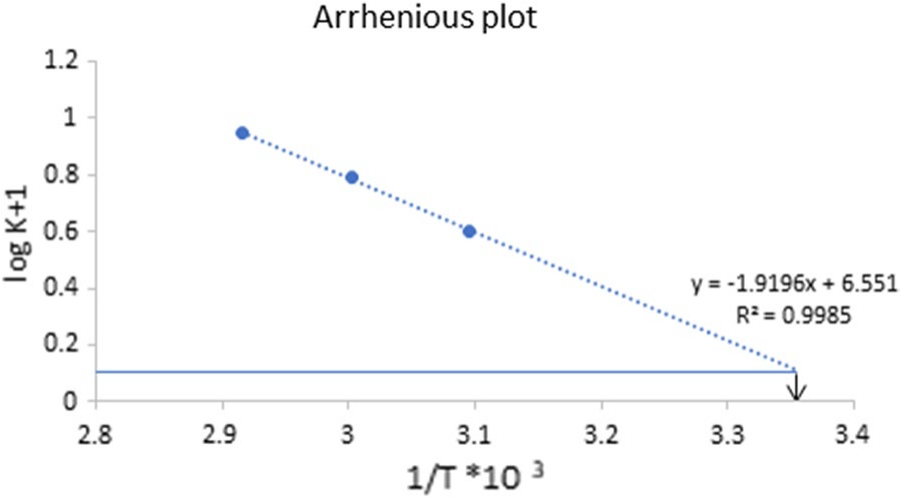
Arrhenius plot for NMV acidic degradation using 2 M HCl

The Arrhenius plot was then derived by plotting the logarithm of degradation rate constant of NMV (k_obs_) against the reciprocals of the absolute temperature [22] as illustrated in Figs. 5 and 6 for the alkaline (0.1 M NaOH) and acidic (2 M HCl) degradation of NMV, respectively. The stability of NMV to the applied degradation conditions was predicted at room temperature by extrapolation to get logK_25_, Figs. 5 and 6. The t_1/2_ value of NMV degradation was then calculated, 0.56 h and 5.39 h for alkaline and acidic degradation, respectively.

### LC–MS-UV study and postulation of degradation mechanisms

Different acidic and alkaline degraded solutions were injected into the LC–MS-UV instrument, and the m/z of the eluted peaks was recorded to investigate the degradation process of NMV. DPs produced under acidic and alkaline conditions with their equivalent m/z values using the scan mode were shown in Additional file 1: Figs. S2–S4.

**Fig. 7:**
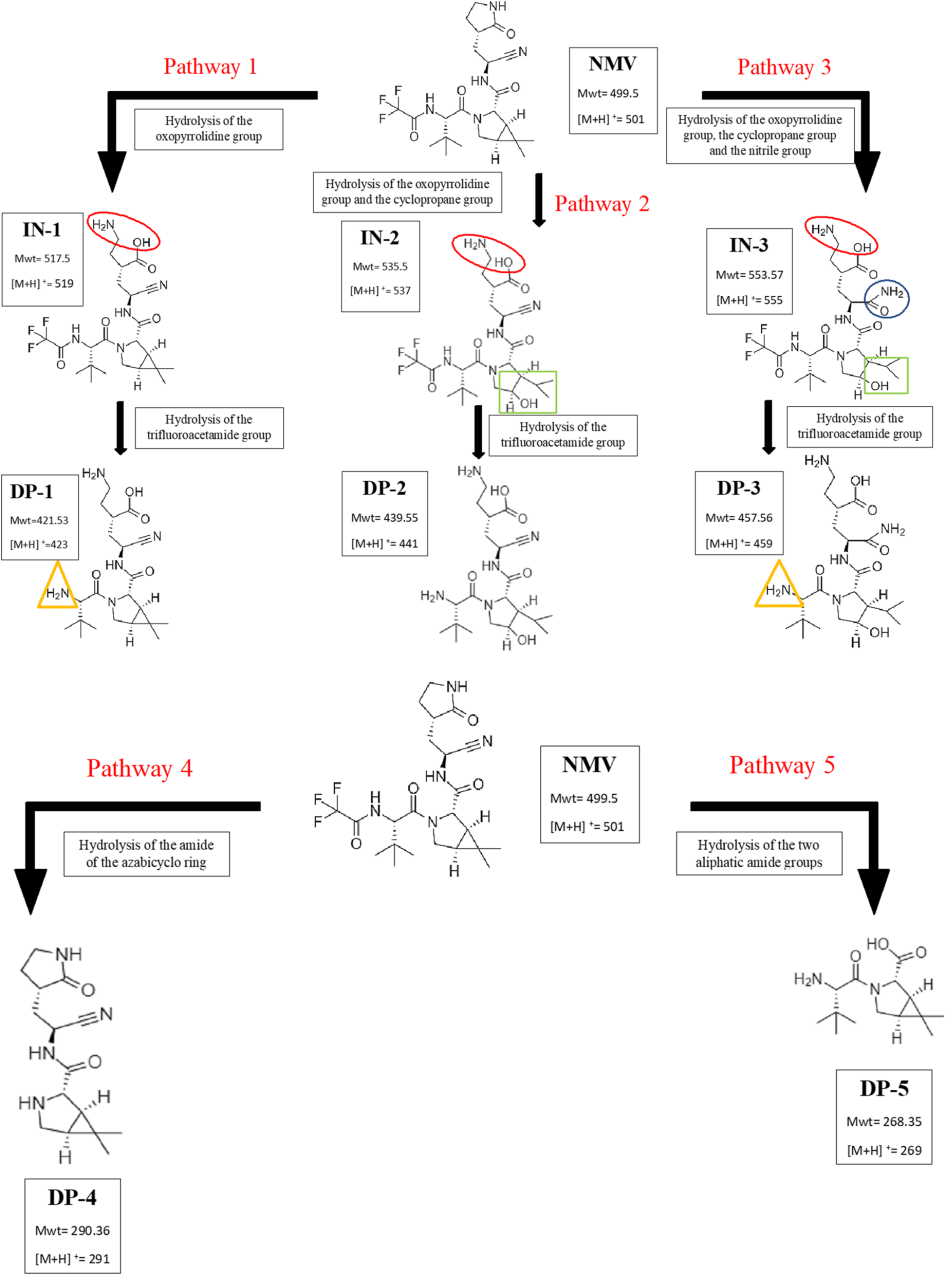
Proposed pathways of the alkaline degradation of NMV

**Fig. 8:**
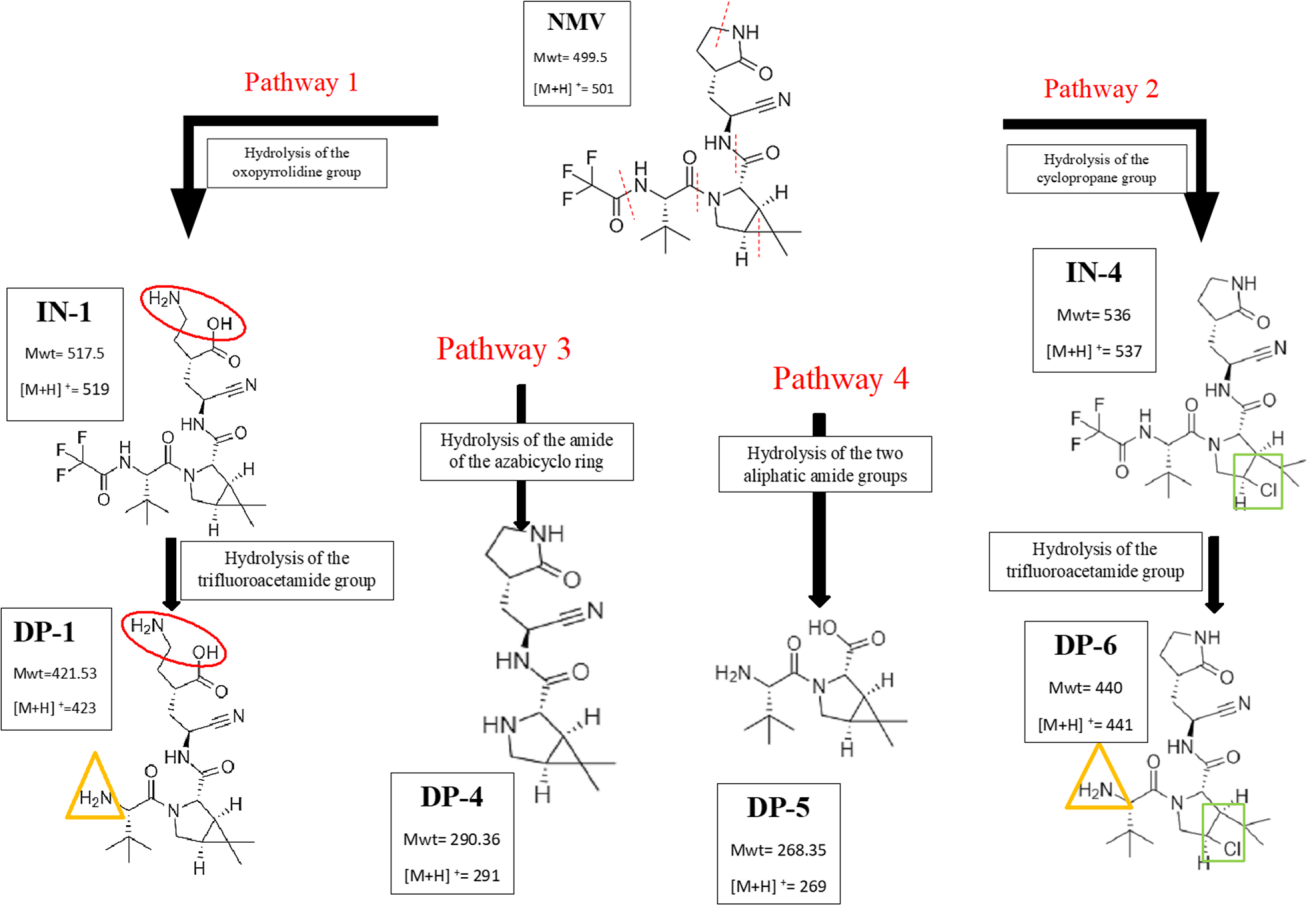
Proposed pathways of the acidic degradation of NMV

Partial alkaline degradation of NMV was accomplished using 0.1 M NaOH at RT for 4 h, mild conditions, with the formation of different intermediates (**INs)** along with NMV peak, m/z 501. The corresponding m/z of the formed **INs** were 519, 537 and 555, Additional file 1: Fig. S3a. **INs** peaks were probably formed by the hydrolysis of amide group in the oxopyrrolidine ring followed by opening of cyclopropane ring and hydrolysis of the nitrile group. The mechanism of base-induced amide hydrolysis was previously studied [23, 24]. In addition, ring opening of cyclopropane using NaOH was studied later by Wong et al. [25]. Due to steric hindrance in NMV structure, it was postulated that the first hydrolysis step was the oxopyrrolidine ring through the addition of one molecule of water to NMV resulting in the formation of **IN-1** with m/z = 501 + 18 = 519, then opening of the cyclopropane ring by the addition of the second water molecule resulting in **IN-2** peak with m/z = 519 + 18 = 537. Afterwards, the nitrile group would undergo hydrolysis to the corresponding amide and an **IN-3** of m/z = 555 (537 + 18) was formed. On the other hand, complete degradation of NMV was practically possible using 0.1 M NaOH at 100 °C for 1 h, hard conditions. In this case, the **INs** peaks disappeared, and other **DPs** peaks, **DP-1, DP-2** and **DP-3** were formed at m/z 423, 441 and 459, Additional file 1: Fig. S3b, as a result of the hydrolysis of the trifluoroacetamide group from **IN-1, IN-2** and **IN-3,** respectively. Moreover, hydrolysis of the amide of the azabicyclo ring resulted in the appearance of **DP-4** with m/z 291. In addition, Hydrolysis of the two aliphatic amide groups yielded **DP-5** with m/z 269. Figure 7 summarizes the proposed pathways of the alkaline degradation of NMV. It is noteworthy to mention that the proposed degradation pathway was different from that postulated by Secreatan et al. [13]. The latter attributed the alkaline degradation to the hydrolysis of the nitrile group and/or trifluoroacetamide group only. However, in the current study, it was proposed that the degradation occurred due to the hydrolysis of the amide group in the oxopyrrolidine ring followed by opening of cyclopropane ring and hydrolysis of the nitrile group. Moreover, hydrolysis of the trifluoroacetamide group and the amide of the azabicyclo ring took place under hard conditions. The difference between the two pathways, our proposed work and ref. [13], could be attributed to the variable conditions used in the degradation, the latter [13] used 1 M NaOH for one week at room temperature (long term stability study) while our current study used 0.1 M NaOH at RT for 4 h and 0.1 M NaOH at 100 °C for 1 h (short term stability study).As per alkaline degradation, mild acidic conditions, 1 M HCl at 90 °C for 1 h, resulted in the formation of **INs** with m/z 519 and 537, Additional file 1: Fig. S4a. The formation of **IN 1** was previously explained under alkaline degradation, while the formation of **IN 2** was attributed to the opening of the cyclopropane ring using HCl. Hard acidic conditions with 2 M HCl at 100 °C for 4 h resulted in the formation of **DPs** peaks, **DP-1, DP-4, DP-5,** and **DP-6** corresponding to m/z 423, 291, 269 and 441, Additional file 1: Fig. S4b. Further hydrolysis of **INs** with removal of the trifluoroacetamide group resulted in the formation of **DP-1** and **DP-6** of m/z 423 and 441, respectively. Additionally, the hydrolysis of the amide of the azabicyclo ring resulted in the formation of **DP-4** with m/z 291, while hydrolysis of the two aliphatic amide groups yielded **DP-5** with m/z 269. The mechanism of acid-induced amide hydrolysis was previously postulated [23, 24]. Moreover, the addition of hydrogen chloride to cyclopropane was discussed by Lee et al. and Lambert et al. [26, 27]. The proposed pathways for acidic hydrolysis of NMV was shown in Fig. 8. As per alkaline hydrolysis, degradation pathways suggested in our study was different from that proposed by Secreatan et al. [13] which attributed the acidic degradation to the hydrolysis of the nitrile group or the trifluoroacetamide group only. Our proposed pathway assumed that the degradation was due to hydrolysis of amide group in the oxopyrrolidine ring or by opening of cyclopropane ring followed by hydrolysis of the trifluoroacetamide group under hard conditions. Moreover, the mechanism of hydrolysis of the azabicyclo ring and the two aliphatic amide groups were differently postulated which could be attributed to the different degradation conditions in the two studies; the published study [13] used 1 M HCl for one week at RT (long term stability study) while the current one used 1 M HCl at 90 °C for 1 h or 2 M HCl at 100 °C for 4 h (short term stability study).

### In-silico toxicity studies

Numerous toxicity end points were predicted using the popular computational toxicity web server, ProTox-II. It featured details about chemical and molecular targets, including computer models, pharmacophores, fragment tolerance, and molecular similarity. ProTox-II models were validated utilising the probability-based CLUSTER cross-validation with selective oversampling [28]. In this study, this database was applied to predict the hazardous potential of NMV and its DPs including cytotoxicity, immunotoxicity, hepatotoxicity, mutagenicity, and carcinogenicity. Table 3 showed that NMV and the proposed DPs (1 to 6) did not exhibit hepatotoxicity, carcinogenicity, mutagenicity, or cytotoxicity with confidence levels ranging from 0.57 to 0.87 [29]. Additionally, the ability of NMV and its DPs to kill 50% of test animals (rodents) after 24 h of exposure (LD50) was used to assess their acute toxicity [28]. NMV, DP-1, DP-2, DP-3, and DP-4 each had an LD50 of 3000 mg/kg, while the LD50 for DP-5 and DP-6 were found to be 5000 and 826 mg/kg, respectively. Based on LD50 (mg/kg) and with reference to ‘classification and labelling of compounds” methodology, NMV and all its DPs, except for DP-6, were classified as being in toxicity class V, while DP-6, being in class IV, is harmful if swallowed [28].

**Table 3:** Predicted probability of toxicity profile of nirmatrelvir and its degradation products (DP-1–DP-6)

Classification	Target	shorthand	Drug	DP-1	DP-2	DP-3	DP-4	DP-5	DP-6	Probability of being inactive
LD 50 (mg/kg)	–	–	3000	3000	3000	3000	3000	5000	826	
Toxicity Class	–	–	5	5	5	5	5	5	4	
Hepatotoxicity	Hepatotoxicity	dili	NA	NA	NA	NA	NA	NA	NA	0.87
Carcinogenicity	Carcinogenicity	carcino	NA	NA	NA	NA	NA	NA	NA	0.60
Immunotoxicity	Immunotoxicity	immuno	NA	NA	NA	NA	NA	NA	NA	0.63
Mutagenicity	Mutagenicity	Mutagen	NA	NA	NA	NA	NA	NA	NA	0.57
Cytotoxicity	Cytotoxicity	Cyto	NA	NA	NA	NA	NA	NA	NA	0.67

### Greenness of the method

The Analytical Eco-Scale [30], the Green Analytical Procedure Index (GAPI) [31], and the innovative Analytical Greenness metric (AGREE) [32] were used in our study to evaluate the degree of greenness of the proposed HPLC–DAD method. Analytical Eco-Scale tool quantifies the method’s degree of greenness [26] based on assigning penalty points (PPs) to each category of the method that deviates from the notion of greenness. The volume of any created waste, the amount of hazardous chemicals that were utilised, and the amount of electricity and energy spent were the categories to be examined. The total determined penalty points were then subtracted from 100. The method is then deemed to be of good greenness if the value is above 50 and outstanding greenness if it is above 75. This tool determined that the estimated value of our proposed method was greater than 75 (Eco-Scale score = 84), Table 4, indicating that it was extremely environmentally friendly with the least adverse effects.

**Table 4:** The penalty points of the proposed chromatographic method according to the analytical Eco-scale

Reagents/Instruments	Penalty points (PPs)
Acetonitrile	3
NaOH	2
HCl	4
H_2_O_2_	4
Energy of HPLC	0
Occupational hazard	0
waste	3
PPs	16
Eco-scale score	84

**Fig. 9:**
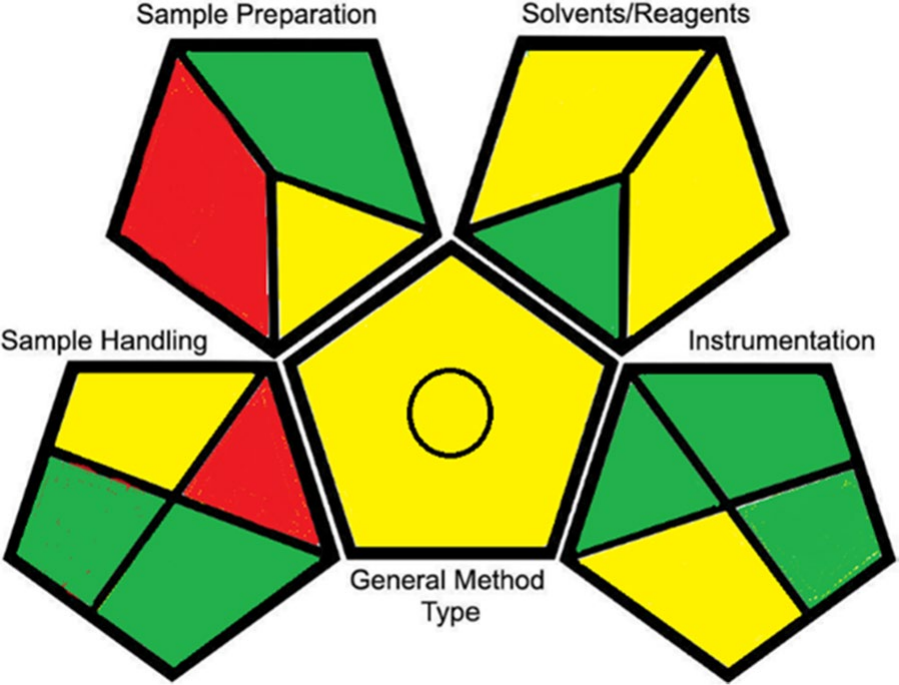
GAPI diagram of the proposed HPLC–DAD method

In addition, our method’s greenness was evaluated using the GAPI and AGREE tools. The GAPI tool assesses the entire analytical procedure, from sample collection and preparation to the conclusion. A vibrant pictogram with 15 elements that refer to 15 assessed process criteria, such as sample preparation and extraction, solvent and reagent properties, energy consumption, waste generation, and occupational dangers, is used to illustrate the method’s eco-friendliness. The non-green characters are given a red tint, and each component is either green (for the greenest element) or yellow (for the least green element). By evaluating our method, it is considered green with 7 green, 6 yellow and 2 red zones, Fig. 9.

**Fig. 10:**
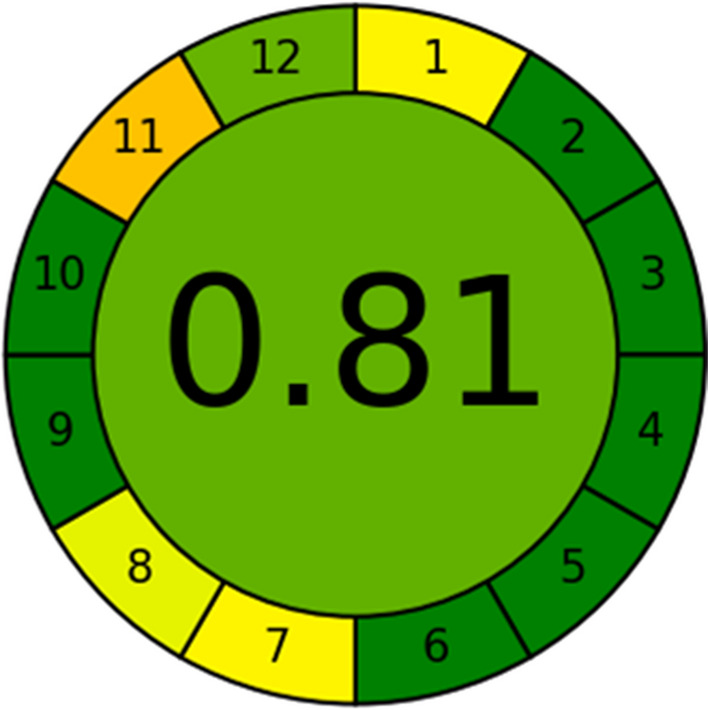
AGREE diagram of the proposed HPLC–DAD method

Lastly, the more recent AGREE metric was used to provide a more detailed, inclusive, and succinct evaluation of the greenness of the proposed method, as well as an interdisciplinary grade based on the fundamental ideas of GAC [33]. This application could be downloaded, and after inputting the 12 characters for the method, it generates a vivid pictogram with 12 segments that are each coloured differently, ranging from deep green to deep red. Additionally, it displays a fractional number in the middle of the circular pictogram; the greener the technique, the closer the final score is to 1. Utilizing this novel strategy led to a more accurate and quantifiable assessment of the methodologies. The AGREE value of the proposed HPLC–DAD method was 0.81 which indicated the greenness of the method as shown in Fig. 10. (The AGREE report was displayed in the Additional file 2).

The aforementioned outcomes showed that the proposed HPLC–DAD method was successfully green.

## Conclusion

A quick, reliable, and precise stability-indicating HPLC–DAD method was developed and validated, *for the first time*, followed by *a kinetics degradation investigation*, to measure NMV in the presence of its forced DPs. It was found that NMV was highly vulnerable to alkali hydrolysis, compared with acid hydrolysis, and that it was stable to conditions that promote oxidative, neutral, and sun-induced degradation. Studying the rates of acid and alkali hydrolysis revealed that the kinetics followed pseudo-first order. Subsequent Arrhenius plot was used to determine the half lifetime and reaction constants of degradation under the studied conditions at room temperature using the proposed method. LC–MS-UV was used to suggest the mechanism of the acid and base degradation pathways. Toxicities of NMV and its DPs were evaluated using ProTox-II and they were determined to be negligibly harmful. Finally, greenness appraisal proved that the proposed HPLC–DAD method was of excellent greenness.

## Supplementary Information

**Additional file 1: Figure S1** Purity plots of NMV peaks obtained from DAD for standard solution, a), and following exposure to different degradation conditions, alkaline, b) acidic, c), oxidative, d) neutral, e), and sunlight, f), degradations. **Figure S2** LC-MS-UV chromatogram of NMV standard solution scanned at different m/z, indicating presence of a peak at m/z 501 for NMV and absence of peaks at other m/z proposed for INs and DPs. **Figure S3** LC-MS-UV chromatograms of MLN using different alkaline stress degradation conditions, mild conditions: 0.1 M NaOH at RT for 4 h indicating presence of a peak at m/z 501 for NMV and m/z for **IN**-1, 2 & 3, a), and hard conditions: 0.1 M NaOH at 100 °C for 1 h, b) indicating absence of a peak at m/z 501 for NMV due to complete degradation and m/z for **DP**-1, 2, 3, 4 & 5. **Figure S4** LC-MS-UV chromatograms of MLN using different acidic stress conditions degradation, mild conditions: 1 M HCl at 90 for 1 h indicating presence of a peak at m/z 501 for NMV and m/z for **IN**-1, 4 & 3, a), and hard conditions: 2 M HCl at 100 °C for 4 h, b) indicating absence of a peak at m/z 501 for NMV due to complete degradation and m/z for **DP**-1, 6, 3, 4 & 5. **Table S1** System suitability parameters for the HPLC-DAD determination of NMV^a^. **Table S2** Intra-day and inter-day accuracy and precision for the determination of NMV. **Table S3** Evaluation of the robustness of the proposed HPLC method for the determination of NMV.

**Additional file 2: **Analytical Greenness report sheet.

## Data Availability

The datasets used and/or analysed during the current study available from the corresponding author on reasonable request.

## Abbreviations

NMV: Nirmatrelvir
HPLC–DAD: High performance liquid chromatography with diode array detector
LOD: Limit of detection
LOQ: Limit of quantification
UFLC-MS: Ultra-fast liquid chromatography-mass spectrometry
DP: Degradation product
IN: Intermediate
GAPI: Green analytical procedure index
AGREE: Analytical Greenness metric
t_1/2_: Half-life times
k_obs_: Reaction rate constants
