# Supplementary material for: Green adherent degradation kinetics study of Nirmatrelvir, an oral anti-COVID-19: characterization of degradation products using LC–MS with insilico toxicity profile
Source: BMC Chem. 2023 Mar 17;17(1):23. doi: 10.1186/s13065-023-00928-z (PMC10020773; doi:10.1186/s13065-023-00928-z)
Supplement: Supplementary file 1 — Additional file 1: Figure S1 Purity plots of NMV peaks obtained from DAD for standard solution, a), and following exposure to different degradation conditions, alkaline, b) acidic, c), oxidative, d) neutral, e), and sunlight, f), degradations. Figure S2 LC-MS-UV chromatogram of NMV standard solution scanned at different m/z, indicating presence of a peak at m/z 501 for NMV and absence of peaks at other m/z proposed for INs and DPs. Figure S3 LC-MS-UV chromatograms of MLN using different alkaline stress degradation conditions, mild conditions: 0.1 M NaOH at RT for 4 h indicating presence of a peak at m/z 501 for NMV and m/z for IN-1, 2 & 3, a), and hard conditions: 0.1 M NaOH at 100 °C for 1 h, b) indicating absence of a peak at m/z 501 for NMV due to complete degradation and m/z for DP-1, 2, 3, 4 & 5. Figure S4 LC-MS-UV chromatograms of MLN using different acidic stress conditions degradation, mild conditions: 1 M HCl at 90 for 1 h indicating presence of a peak at m/z 501 for NMV and m/z for IN-1, 4 & 3, a), and hard conditions: 2 M HCl at 100 °C for 4 h, b) indicating absence of a peak at m/z 501 for NMV due to complete degradation and m/z for DP-1, 6, 3, 4 & 5. Table S1 System suitability parameters for the HPLC-DAD determination of NMVa. Table S2 Intra-day and inter-day accuracy and precision for the determination of NMV. Table S3 Evaluation of the robustness of the proposed HPLC method for the determination of NMV. [file 13065_2023_928_MOESM1_ESM.docx]

Supplementary figures


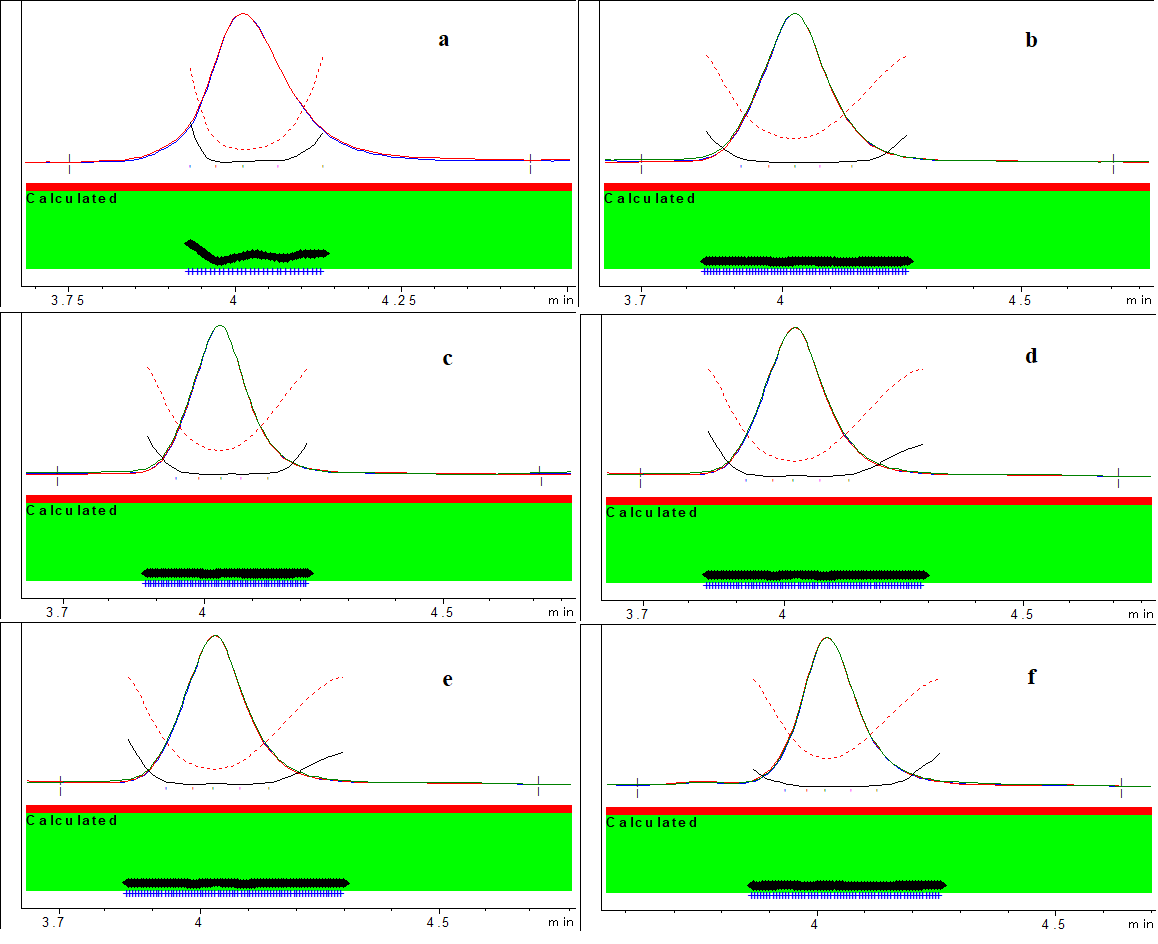


Figure S1: Purity plots of NMV peaks obtained from DAD for standard solution, a), and following exposure to different degradation conditions, alkaline, b) acidic, c), oxidative, d) neutral, e), and sunlight, f), degradations.


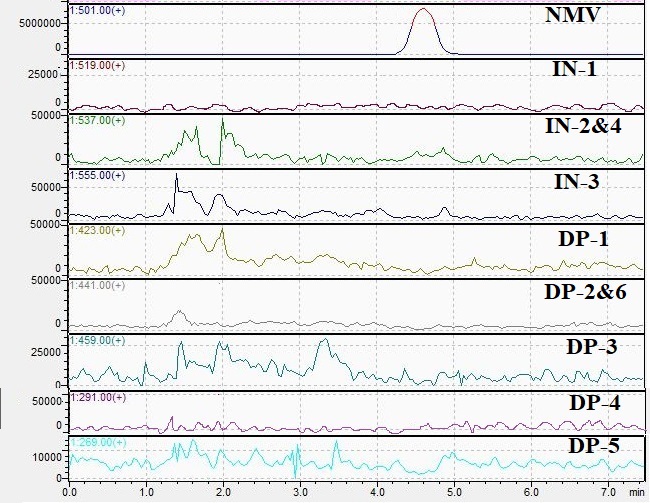


Figure S2: LC-MS-UV chromatogram of NMV standard solution scanned at different m/z, indicating presence of a peak at m/z 501 for NMV and absence of peaks at other m/z proposed for INs and DPs.


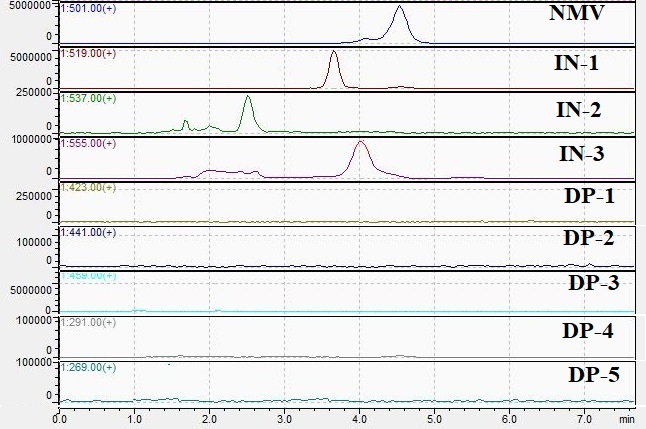

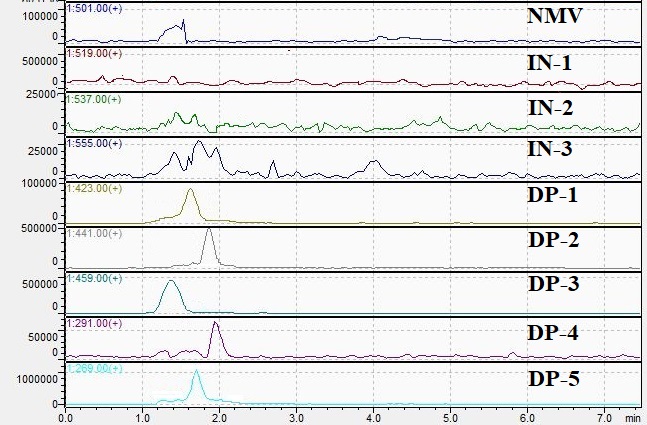


b)

a)

Figure S3: LC-MS-UV chromatograms of MLN using different alkaline stress degradation conditions, mild conditions: 0.1 M NaOH at RT for 4 h indicating presence of a peak at m/z 501 for NMV and m/z for **IN**-1, 2 & 3, a), and hard conditions: 0.1 M NaOH at 100 °C for 1 h, b) indicating absence of a peak at m/z 501 for NMV due to complete degradation and m/z for **DP**-1, 2, 3, 4 & 5.


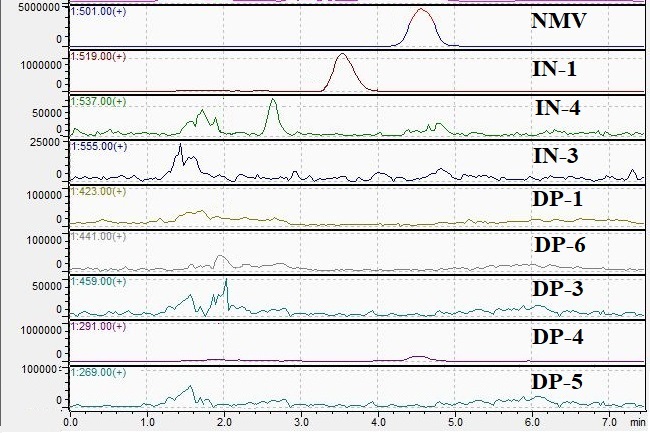

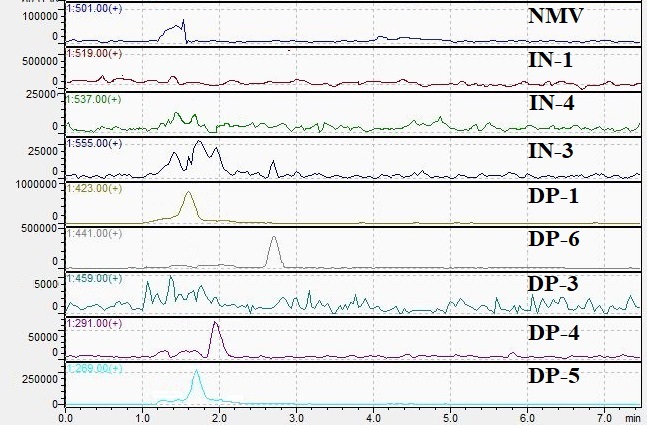


b)

a)

Figure S4: LC-MS-UV chromatograms of MLN using different acidic stress conditions degradation, mild conditions: 1 M HCl at 90 for 1 h indicating presence of a peak at m/z 501 for NMV and m/z for **IN**-1, 4 & 3, a), and hard conditions: 2 M HCl at 100 °C for 4 h, b) indicating absence of a peak at m/z 501 for NMV due to complete degradation and m/z for **DP**-1, 6, 3, 4 & 5.

Additional file tables

Table S1. System suitability parameters for the HPLC-DAD determination of NMV ^a^.

| **Parameters** | **NMV** |
| --- | --- |
| **t_R_ ± SD (min) ^b^** | 4.01 ± 0.01 |
| **Capacity factor (k')** | 3.02 |
| **Theoretical plates (N)** | 7850 |
| **Resolution (R_s_) ^c^** | 2.22 |
| **Tailing factor (T_f_)** | 0.97 |
| ^a^ System suitability recommendations: k' (1-10), N > 2000, R_s_ > 2 and T_f_ (0.8 -1.2) [17]  ^b^ Average t_R_ ± SD of three determinations.  ^c^ Resolution between the drug and the nearest DP. | |

Table S2. Intra-day and inter-day accuracy and precision for the determination of NMV.

| **Concentration µg/mL** | % Recovery ± SD^a^ | **RSD (%)^b^** | **E_r_ (%)^c^** |
| --- | --- | --- | --- |
| 1. Intra-day accuracy and precision | | | |
| 6 | 101.90±1.74 | 1.74 | 1.90 |
| 40 | 99.95±1.25 | 1.24 | -0.05 |
| 400 | 100.90±0.26 | 0.27 | 0.90 |
| 1. Inter-day accuracy and precision | | | |
| 6 | 98.16±1.91 | 1.90 | -1.84 |
| 40 | 100.23±0.39 | 0.39 | 0.23 |
| 400 | 100.13±0.15 | 0.15 | 0.13 |
| **^a^** Mean ± standard deviation for three determinations**.**  **^b^** % Relative standard deviation.  **^c^** % Relative error. | | | |

Table S3. Evaluation of the robustness of the proposed HPLC method for the determination of NMV.

| **Parameters** | **Mean % recovery ± SD ^a^** | **RSD% ^b^** | **t_R_ ± SD ^c^** |
| --- | --- | --- | --- |
| **Ratio of organic modifier mobile phase (50% ± 2%)** | 100.15±0.06 | 0.06 | 4.01±0.21 |
| **Wavelength of determination (225 ± 1 nm)** | 98.64±0.10 | 0.10 |  |
| **pH of buffer (5 ± 0.2 pH units)** | 100.01±0.07 | 0.07 | 4.01±0.05 |
| **^a^** Mean of percentage recovery of peak area of NMV of concentration 50 µg/mL at the studied parameters ± SD.  **^b^** Percentage relative standard deviation of % recovery of NMV at the studied parameters.  **^c^** Mean of retention time of NMV at the studied parameters **±** SD. | | | |
