# Supplementary material for: Green adherent degradation kinetics study of Nirmatrelvir, an oral anti-COVID-19: characterization of degradation products using LC–MS with insilico toxicity profile
Source: BMC Chem. 2023 Mar 17;17(1):23. doi: 10.1186/s13065-023-00928-z (PMC10020773; doi:10.1186/s13065-023-00928-z)
Supplement: Supplementary file 2 — Additional file 2: Analytical Greenness report sheet. [file 13065_2023_928_MOESM2_ESM.pdf]

# Analytical Greenness report sheet

11/07/2022 10:48:24

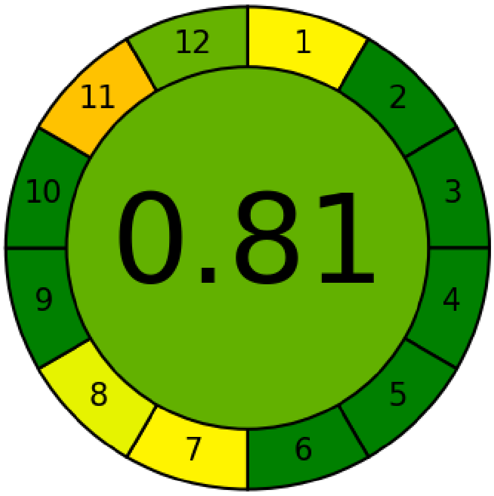


| Criteria | Score | Weight |
|---|---|---|
| 1. Direct analytical techniques should be applied to avoid sample treatment. | 0.48 | 2 |
| 2. Minimal sample size and minimal number of samples are goals. | 1.0 | 2 |
| 3. If possible, measurements should be performed in situ. | 1.0 | 2 |
| 4. Integration of analytical processes and operations saves energy and reduces the use of reagents. | 1.0 | 2 |
| 5. Automated and miniaturized methods should be selected. | 1.0 | 2 |
| 6. Derivatization should be avoided. | 1.0 | 2 |
| 7. Generation of a large volume of analytical waste should be avoided, and proper management of analytical waste should be provided. | 0.48 | 2 |
| 8. Multi-analyte or multi-parameter methods are preferred versus methods using one analyte at a time. | 0.55 | 2 |
| 9. The use of energy should be minimized. | 1.0 | 2 |
| 10. Reagents obtained from renewable sources should be preferred. | 1.0 | 2 |
| 11. Toxic reagents should be eliminated or replaced. | 0.38 | 2 |
| 12. Operator's safety should be increased. | 0.8 | 2 |
